# Freezing and thawing cycles affect nitrous oxide emissions in rain-fed lucerne (*Medicago sativa*) grasslands of different ages

**DOI:** 10.7717/peerj.12216

**Published:** 2021-10-05

**Authors:** Yuan Li, Yuying Shen, Tao Wang

**Affiliations:** 1 State Key Labouratory of Grassland Agro-ecosystems of Lanzhou University, Lanzhou, China; 2 College of Pastoral Agriculture Science and Technology, Lanzhou University, Lanzhou, China

**Keywords:** Denitrification, Loess Plateau, *Medicago sativa*, Nitrous oxide, Soil temperature, Freeze/thaw cycle, Loess Plateau

## Abstract

Lucerne (*Medicago sativa* L.) is a major component of the crops used in dry-land farming systems in China and its management is associated with notable nitrous oxide (N_2_O) emissions. A high proportion of these emissions is more likely to occur during periods when the soil undergoes freezing and thawing cycles. In this study, the effects of freeze/thaw cycles on N_2_O emissions and related factors were investigated in lucerne grasslands. The hypothesis was tested whether increased emissions resulted from a disruption of nitrification or denitrification caused by variations in soil temperatures and water contents. Three days (3 × 24 h) were chosen, where conditions represented freezing and thawing cycles. N_2_O emissions were measured for a fallow control (F) and two grasslands where lucerne had been cultivated for 4 and 11 years. Soil temperature, soil water content, soil microbial biomass carbon (MBC), soil microbial biomass nitrogen (MBN), soil ammonium nitrogen (NH_4_^+^-N), and soil nitrate nitrogen (NO_3_^−^-N) contents were measured. Moreover, the quantities of soil nitrification and denitrification microbes were assessed. Variations in N_2_O emissions were strongly affected by freeze/thaw cycles, and emissions of 0.0287 ± 0.0009, 0.0230 ± 0.0019, and 0.3522 ± 0.0029 mg m^−2^ h^−1^ were found for fallow, 4-year-old, and 11-year-old grasslands, respectively. Pearson correlation analyses indicated that N_2_O emissions were significantly correlated with the soil water content, temperature, NH_4_^+^-N content, and the number of nitrosobacteria and denitrifying bacteria at a soil depth of 0–100 mm. The numbers of nitrosobacteria and denitrifying bacteria correlated significantly with soil temperature at this soil depth. MBN and soil NH_4_^+^-N contents correlated significantly with soil water content at this depth. Principal component analysis highlighted the positive effects of the number of denitrifying bacteria on N_2_O emissions during the freeze/thaw period. Furthermore, soil temperature and the number of nitrosobacteria at the tested soil depth (0−100 mm) also played a significant role. This shows that soil freeze/thaw cycles strongly impacted both N_2_O emissions and the diurnal range, and the number of denitrifying bacteria was mainly influenced by soil temperature and soil NH_4_^+^-N content. The number of denitrifying bacteria was the dominant variable affecting N_2_O emissions from lucerne grasslands during the assessed soil freeze/thaw period on the Loess Plateau, China.

## Introduction

Nitrous oxide (N_2_O) is a potent greenhouse gas in the atmosphere and contributes to global warming because of its high global warming potential (GWP-100 y) of 296, relative to the value of 1 for CO_2_ (IPCC, 2013). Mitigating N_2_O emissions generated by the agricultural sector is a major challenge, as agriculture contributes ~60% of the global N_2_O emissions (IPCC, 2013).

Soil freezing and thawing cycles that occur mainly in spring, autumn, and during mild winter periods have been shown to cause substantial physical and biological changes in soils, inducing pulses in N_2_O emissions (Kariyapperuma et al., 2011; Wang et al., 2017). Previous studies have observed these phenomena in both laboratory and field experiments. For instance, a laboratory investigation assessed two soils with various freezing and thawing rates and found high soil N_2_O emissions during both freezing and thawing periods (Pelster et al., 2019). Another study reported that N_2_O emissions from a temperate semi-arid steppe were strongly affected by freeze-thawing cycles (Wang et al., 2017). A synthetic study indicated that neglecting these freeze–thaw emissions leads to an underestimation of global agricultural N_2_O emissions by 17% to 28% (Wagner-Riddle et al., 2017). Studies also indicated that N_2_O emissions driven by soil freeze/thaw cycles contribute strongly to annual N_2_O emission budgets (Koponen & Martikainen, 2004; Butterbach-Bahl & Wolf, 2017; Cambareri et al., 2017). To better understand the underlying mechanisms, the contribution of N_2_O emissions during soil freeze/thaw cycles needs to be quantified.

Three aspects contribute to the regulation of N_2_O emissions during freeze/thaw cycles: soil carbon (C) and nitrogen (N) contents, the temperature and water content of soil, and soil microbial activity. Increasing the available C and N contents for microorganisms in soils enhances N_2_O emissions (Song et al., 2017; Li et al., 2021). For example, freeze/thaw cycles could increase the soil C content by releasing physically protected soil organic material, and soil perturbation could increase soil N content by accelerating N mineralization (Christensen & Christensen, 1991). Soil freeze/thaw cycles also affect the dynamics of soil microbial biomass carbon (MBC) and soil microbial biomass nitrogen (MBN) (Gou et al., 2015; Song et al., 2017). Soil temperature and water content influence N_2_O emissions directly by affecting the rates of nitrification and denitrification, soil aeration, and the availability of both C and N in soil (Luo et al., 2013; Li et al., 2021). The phase transition of soil water during freeze/thaw cycles acts as a microclimatic perturbation, which may play a prominent role in increasing N_2_O emissions (Müller et al., 2002; Wu et al., 2013; Congreves et al., 2018; Pinto et al., 2021). An *in-situ* experiment study in the temperate semi-arid steppe indicated that soil temperature is influenced by seasonal thawing (Wang et al., 2017). N_2_O emissions induced by freeze/thaw cycles resulted from stressing initiated high microbial activity related to nitrification or denitrification (Goreau, Kaplan & Wofsy, 1980; Christensen & Christensen, 1991; Holtan-Hartwig, Dörsch & Bakken, 2002). Research on the semiarid northern Great Plains (USA) showed that denitrification is more prominent during freeze/thaw cycles (Dusenbury et al., 2008). However, to date, the N_2_O emissions in response to freeze/thaw cycles in lucerne grasslands have not received sufficient attention. This is important, as biological nitrogen fixation provides additional substrates for nitrification and denitrification (Rochette & Janzen, 2005).

Lucerne (*Medicago sativa*) is an important crop in dryland farming systems of the Loess Plateau, China, and has been shown to produce high N_2_O emissions (Bremner, 1997; IPCC, 2013; Ning et al., 2020). The Loess Plateau occupies more than 70% of the farmland in north-western China and is characterized by near-surface soil freeze/thaw cycles in early spring and late autumn (Han, Tsunekawa & Tsubo, 2010). However, most studies measuring N_2_O emissions from lucerne have been conducted during the growing season, while only few studies focused on freeze/thaw cycles outside of controlled conditions in laboratory experiments (Müller et al., 2002; Rochette & Janzen, 2005; Chapuis-lardy et al., 2007; Wu et al., 2013). Studies conducted in the field are therefore needed to quantify the effect of freeze/thaw cycles on the soil physical properties and microbial activities that affect N_2_O emissions (Henry, 2007).

In the present study, the hypothesis was tested whether N_2_O emissions during freeze/thaw cycles respond to variations in the rates of nitrification or denitrification, resulting from the dynamics of soil temperature and water content. Soil available C and N contents as well as the rhizobium are related to the age of lucerne grasslands (Hong, 2009; Wang et al., 2014b). Therefore, N_2_O emissions from two lucerne grasslands with different ages (*i.e*., 4-years-old and 11-years-old) on the Loess Plateau were also investigated. The specific objectives were to (1) quantify the effects of freeze/thaw cycles on N_2_O emissions and (2) determine if changes in emissions were attributable to changes in soil C and N availability, soil temperature, soil water content, or increasing rates of nitrification and/or denitrification.

## Materials & methods

### Site description

The study was undertaken at the Qingyang Loess Plateau Research Station of Lanzhou University, China (lat. 35°39′N, long. 107°51′E, elevation above sea level 1,297 m) during 2013–2014. The climate is a typical cool continental climate, with mean annual precipitation of 548 mm, with the highest abundance of rainfall from late summer (July) to autumn (September). The average annual temperature ranges from 8 °C to 10 °C. The mean temperatures in the warmest (July) and coldest (January) months are 21.3 °C and −5.3 °C, respectively. The growing season extends from spring (March) to mid autumn (October) for about 255 d with 110 frost-free days on average. The soil type is Heilu soil, which is defined as silty loam soil based on the U.S. classification system (Entisol of FAO classification). The properties of sampled soils at the start of the measurement periods are presented in Table 1.

**Table 1:** Measurements of soil organic carbon (SOC), total nitrogen (N) and soil pH in the soil for the F (fallow control) and 4-year old and 11-year old lucerne grasslands at different depths. Data shown are mean ± standard deviation, *n* = 3.

Site	Depth (mm)	SOC (mg g^−1^)	*N* (mg g^−1^)	C/N	pH
F	0–100	10.23 ± 0.61	1.11 ± 0.01	9.22 ± 2.20	7.53 ± 0.08
4-year		13.49 ± 0.77	1.62 ± 0.11	8.32 ± 2.78	7.22 ± 0.05
11-year		16.45 ± 1.37	1.82 ± 0.11	9.04 ± 4.11	7.13 ± 0.12
F	100–200	8.54 ± 0.47	0.88 ± 0.06	9.71 ± 3.19	–
4-year		11.20 ± 0.62	1.26 ± 0.08	8.90 ± 2.41	–
11-year		13.64 ± 1.17	1.39 ± 0.09	9.80 ± 4.50	–
F	200–300	7.52 ± 0.47	0.89 ± 0.04	8.46 ± 1.14	–
4-year		8.42 ± 0.42	0.90 ± 0.09	9.32 ± 0.85	–
11-year		8.62 ± 0.38	0.94 ± 0.07	9.17 ± 1.89	–

### Experimental design and N_2_O emissions measurements

The experiment was a randomized block design comprising two treatments with 4-year-old lucerne and 11-year-old lucerne (*Medicago sativa* L. cv Longdong) cultivation, which were planted following maize crops (*Zea mays* L.). Afterwards, neither of these two lucerne grasslands was further reseeded. A fallow plot (F) with no retention of previous crop residue and no soil disturbances was used as control. Plot dimensions were 3 × 4 m and three replicates were used. Both lucerne grasslands were drilled sown, with 0.3 m row spacing and a sowing rate of 15 kg ha^−1^. Lucerne grasslands were rain-fed and ammonium phosphate was applied at a rate of 302 kg ha^−1^ at the recovering (or regreening) stage each year. Lucerne was cut twice each year as forage when the grassland reached the flowering stage, which is the typical cultivation mode in this region (Shen et al., 2009).

N_2_O emissions were measured in the autumn-winter period (28 November 2013), the winter-spring period (6 March 2014), and during a mild period in winter (7 January 2014). These 3 days were selected as they represent typical temperature fluctuations during a freeze/thaw for across the soil profile, cycle amplitude, and minimum temperature of sub-surface soil (Henry, 2007; Kariyapperuma et al., 2011). Emissions were measured using a N_2_O/CO near-infrared gas analyzer (Model DLT-100; Los Gatos Research, Inc., San Jose, CA, USA) connected to automated sampling chambers (diameter 350 mm, height 600 mm, model SC-03, LI-CA, China) placed in each plot. Sampling chambers were fitted with a small fan mounted inside the lid to maintain well-mixed conditions. The chambers were distributed randomly in each plot and were embedded with chamber collars, which were pressed into the soil to a depth of 10 cm two weeks prior to the first gas measurement (Fig. S1). For one replicate of N_2_O measurement, *e.g*., the chamber under F was closed while chambers under 4-year-old and 11-year-old grasslands remained open. Gas samples of each plot were taken every 1 min in sequential order; hence, one replicate of N_2_O measurement from F, 4-year-old, and 11-year-old grasslands required a total of 20 min. The sampling sequence was repeated a further two times, yielding a total of three replicates over a 60 min period. Automated chambers were sealed airtight during the sampling procedure by two lids that closed and opened *via* an air pump.

Data were logged by a soil flux system (MCC-1-8, LI-CA, China). The main problem observed during these experiments was related to chamber sealing in the field condition; thus, the despike function in the ‘oce’ package (Kelley, 2018) was used to identify N_2_O spikes with respect to a “reference” time-series. Spikes with N_2_O values of 2 min before and after the time point when a spike occurred were replaced with a reference value. Eventually, out of 648 emissions, 69% were real observations, and 21% were replaced by a reference value. The remaining 10% had no values. Daily N_2_O emissions were calculated and integrated over time to obtain cumulative emissions over the study period.

### Soil measurements and analysis

During each sampling period, soil temperature and volumetric water contents were measured hourly by probes placed close to each chamber, installed at depths of 50, 150, and 250 mm. Soil samples, at depths of 0–100, 100–200, and 200–300 mm, were taken at five sites near each chamber using a 50 mm diameter gouge auger at around 10:00 am. Soil samples were placed in airtight plastic bags, transported to the laboratory, and stored temporarily at 4 °C in the dark (for less than 1 week). Fresh soil was analyzed for MBC, MBN, and mineral N (NH_4_^+^-N and NO_3_^−^-N). Total organic carbon (SOC), total N (TN), and soil pH were analyzed using air-dried soil.

SOC was measured using dichromate oxidation and total N according to the Kjeldahl method (Kjeltec™ 8400; FOSS, Sweden). Soil mineral nitrogen (NO_3_^−^ and NH_4_^+^) were extracted with 1 M KCl and measured with a colorimetric continuous flow analyzer (Model FIAstar 5000; FOSS, Sweden). Soil pH (1:2.5 soil-water paste) was measured using electrometry with a pH electrode (Model PSH-25C; JTONE, Shantou, China).

Soil MBC and MBN were determined using the fumigation-extraction method (Brookes et al., 1985; Vance, Brookes & Jenkinson, 1987). One portion of fresh soil (20 g oven-dried equivalent) was shaken with 80 mL of 0.5 M K_2_SO_4_ for 30 min, filtered through quantitative filter paper (medium-speed), and frozen at −20 °C prior to analysis. Simultaneously, another soil sample was fumigated with ethanol-free chloroform for 24 h at 25 °C and then, the extraction was undertaken as above. Dichromate oxidation was used to determine total organic extracts. The concentration of N in the extracted sample was measured colorimetrically with a continuous flow analyzer (Model FIAstar 5000; FOSS, Sweden). MBC and MBN were calculated as the difference between SOC and TN in the fumigated and non-fumigated extracts using K_EC_ = 0.38 and K_EN_ = 0.54, respectively (Brookes & Joergensen, 2005).

### Detection of microbial numbers

To calculate the most probable numbers (MPN), the technique of dilution and incubation of replicated cultures across several serial dilution steps was used (Oblinger & Koburger, 1975). In brief, an aliquot of 10 g (fresh weight) of mixed material from a soil depth of 0–100 mm was suspended in 90 mL of autoclave-sterilized distilled water in a 250 mL flask (a dilution of 10^−1^). Soil suspensions were shaken for 30 min on a shaker (130 rpm) at room temperature (20 °C). The diluted soil samples were allowed to settle for 5 min; then, 1 mL was decanted by a sterile pipette into 10 mL dilution tubes containing 9 mL sterilized distilled water, yielding a 10^−2^ dilution. This was repeated down to a dilution of 10^−5^, to obtain a series of 10-fold soil dilutions from 10^−1^ to 10^−6^. One mL of each soil dilution was mixed with modified Stephenson media (Sylvia et al., 2005). The tubes were then closed with a sterile permeability sealing membrane (for denitrifying microbes, sealing film was used) and incubated for a period of 14 days at 25 °C and 65% relative humidity in the dark.

In summary, in this technique, replicate portions of the original sample were cultured to determine the presence or absence of microorganisms in each portion. After subdividing the sample, each portion was incubated in a specific nutrient milieu that selects certain organisms or groups of organisms. At the end of the appropriate incubation period, each portion was checked for presence or absence of growth. The MPN of target organisms present in the original sample is either calculated or determined by consulting a table (Williams & Busta, 1999). Measurements of MPN were used to provide an indication of the ability of soil microbes to utilize various nitrogen sources, following a procedure adapted from Sylvia et al. (2005).

### Statistical analyses

Soil N_2_O emissions at each hour, soil water content, soil temperature, MBC, MBN, mineral-N (both NH_4_^+^-N and NO_3_^−^-N), microbial numbers, and cumulative N_2_O emissions were analyzed statistically using Genstat (version 17.0, VSN International Ltd., Hemel Hempstead, UK) (Cambareri et al., 2017). ANOVA was applied for testing significant differences between the ages of lucerne grassland (4-year-old and 11-year-old grasslands) during the same period. Comparisons between F, 4-year-old, and 11-year-old grasslands were made using the least significant difference (LSD) test at 5%. For MPN comparison, treatment was set as fixed factors and the sampling date was set as a random factor.

Principal component analysis (PCA) was performed to reduce the number of variables in the correlation matrix obtained from Pearson correlation analysis, and to select the most discriminating variables affecting N_2_O emissions. Normality and homogeneity of variances were assessed with the Shapiro–Wilk and Levene and Barlett tests.

## Results

### N_2_O emissions

**Figure 1:**
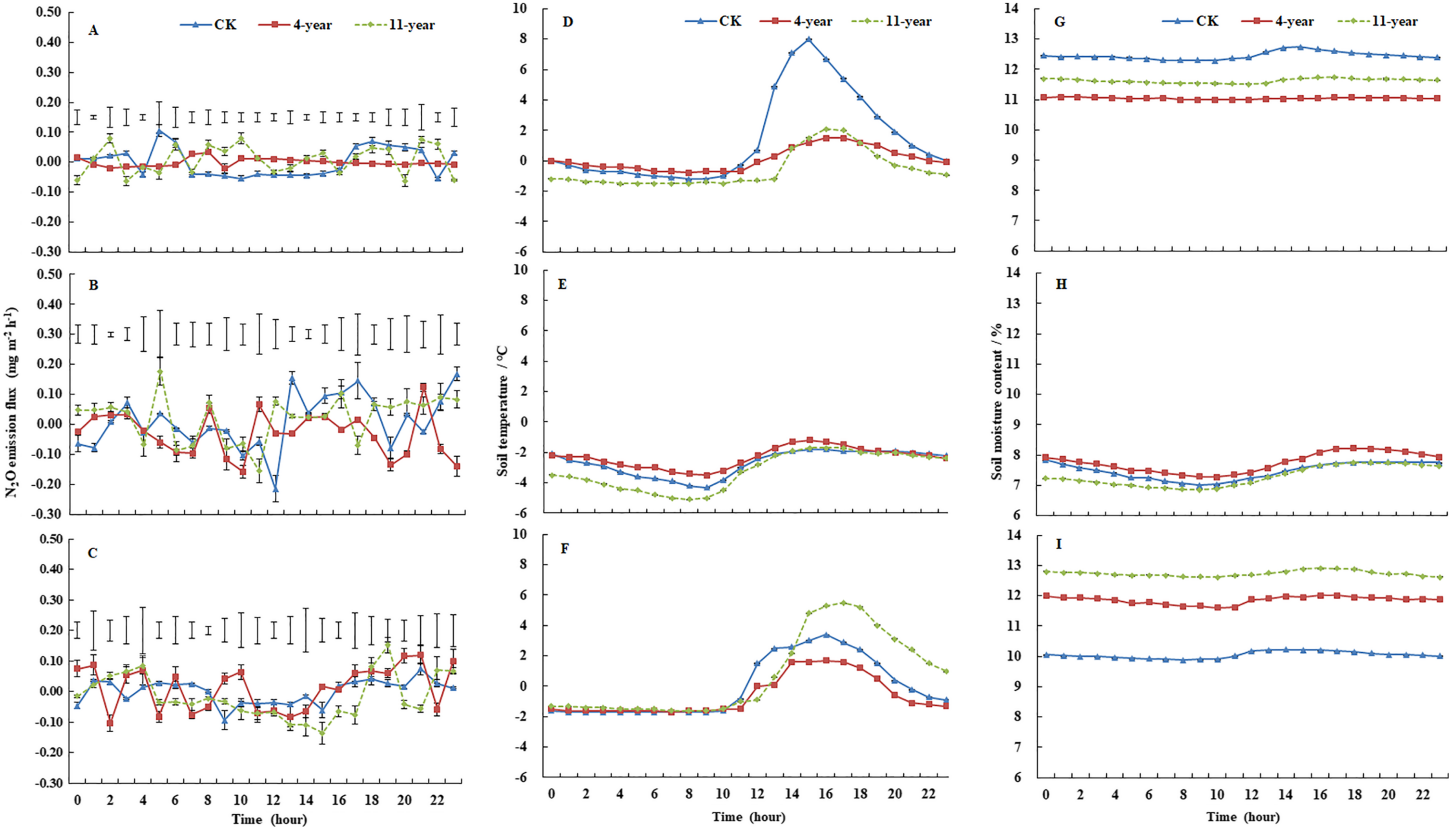
Dynamics in soil temperatures, water content and N_2_O fluxes (mean ± standard deviation, *n* = 3) at 0–100 mm. For the F (fallow control), 4-year old and 11-year old lucerne grasslands on 28 November 2013 (A, D and G), 7 January 2014 (B, E and H) and 6 March 2014 (C, F and I). Bars represent the least significant difference LSD (*P* = 0.05) values for N_2_O fluxes.

Mean (± standard deviation) N_2_O emissions from the F, 4-year-old, and 11-year-old grasslands for each freeze/thaw cycle were 0.0287 ± 0.0009, 0.0230 ± 0.0019, and 0.3522 ± 0.0029 mg m^−2^ h^−1^, respectively (Figs. 1A, 1B and 1C). On 28 November, the mean N_2_O emissions ranked 11-year-old grassland > F > 4-year-old grassland, and emissions of the 11-year-old grassland ranged from −0.0014 to 0.0879 mg m^−2^ h^−1^ (Fig. 1A, *P* < 0.05). On 7 January, the variability was highest for F plots. The average emissions ranked 11-year-old grassland > F > 4-year-old grassland with values of 0.0477, 0.0439, and 0.0173 mg m^−2^ h^−1^, respectively (Fig. 1B, *P* < 0.05). On 6 March, the daily variability in N_2_O emissions was highest for the 11-year-old grassland (*P* < 0.05), which ranged from −0.0007 to 0.1519 mg m^−2^ h^−1^ (Fig. 1C). Variability of N_2_O emissions was small for the three treatments during the time from 12:00 to 16:00 when mean emissions ranked F > 4-year-old grassland > 11-year-old grassland with values of 0.0086, 0.0066, and 0.0005 mg m^−2^ h^−1^, respectively (*P* < 0.05). Compared with the daily range of N_2_O emissions in January and March, the observed range was much lower in November, especially during the hours from 12:00 to 14:00.

Cumulative N_2_O emissions were 7.56, −16.98, and 9.61 mg N m^−2^ for F, 4-year-old grassland, and 11-year-old grassland, respectively. Cumulative N_2_O emissions from F and 11-year-old grasslands showed no difference, and were significantly higher than that of 4-year-old grasslands.

### Soil temperatures and soil water content

Regarding soil temperature for the three treatments at a depth of 0–100 mm (Figs. 1D, 1E and 1F), the F plot showed the highest variation on 28 November, with values ranging from −1.2 °C to 8 °C, and values for the three treatments were below zero from 01:00 to 11:00 (Fig. 1D). On 7 January, the soil temperature of the three treatments was below zero and showed low variability (Fig. 1E). On 6 March, soil temperatures were below zero during the period from 00:00 to 11:00, and the variation was highest for the 11-year-old grassland, where it ranged from −1.6 °C to 5.5 °C (Fig. 1D).

On 28 November, the values of soil water content at a depth of 0–100 mm ranged from 12.20% to 12.74%, 11.51% to 11.74%, and 10.99 to 11.08% for the F, 4-year-old, and 11-year-old grasslands, respectively (Fig. 1G). On 7 January, soil water contents ranged from 7.01% to 7.84%, 6.85% to 7.75%, and 7.28% to 8.22% for the F, 4-year-old, and 11-year-old grasslands, respectively (Fig. 1H). On 6 March, soil water content followed the order of 11-year-old > 4-year-old > F (*P* < 0.05), with values of 12.20% to 12.74%, 11.61% to 12.01%, and 12.62% to 12.91%, respectively (Fig. 1I).

### MBN and MBC concentrations

The values of MBN and MBC decreased with increasing soil depth (Table 2). MBN and MBC values in the topsoil (0–100 mm) were higher than at a depth of 100–200 mm (*P* < 0.05), and both were higher than those at a depth of 200–300 mm (*P* < 0.05). Values of MBN and MBC were higher for the 11-year-old grassland than those for the 4-year-old grassland (*P* < 0.05). Values of MBN in the topsoil (0–100 mm depth) for the lucerne grassland were higher compared with values for F (*P* < 0.05). MBN decreased from the 28 November to the 7 January, and then increased to 6 March, while values of MBC gradually decreased from November to March.

**Table 2:** Microbial biomass carbon (MBC) and nitrogen (MBN) (mg kg^−1^) at different soil depths for the three measurement periods for the F (fallow control), 4 and 11-year old lucerne grasslands. The data shown are mean ± standard deviation; *n* = 3.

Date	Treatment Depth (mm)	MBN			MBC		
		0–100	100–200	200–300	0–100	100–200	200–300
28 November 2013	F	12.30 ± 3.50	5.97 ± 0.90	3.71 ± 0.57	259.46 ± 62.69	75.33 ± 25.17	54.52 ± 3.45
	4-year	16.18 ± 3.18	7.88 ± 2.43	5.03 ± 1.64	313.19 ± 44.13	99.84 ± 34.95	82.23 ± 19.23
	11-year	17.35 ± 1.88	8.49 ± 1.74	5.13 ± 1.95	366.95 ± 95.76	100.22 ± 33.33	91.15 ± 18.13
7 January 2014	F	9.27 ± 1.58	6.97 ± 0.44	3.81 ± 0.94	191.56 ± 45.82	84.08 ± 14.75	53.74 ± 16.29
	4-year	14.11 ± 1.27	8.77 ± 1.82	4.87 ± 0.38	267.29 ± 59.37	94.48 ± 20.19	78.32 ± 12.46
	11-year	15.46 ± 2.47	9.32 ± 1.46	4.97 ± 1.92	294.84 ± 49.13	95.32 ± 16.27	85.01 ± 10.61
6 March 2014	F	13.89 ± 3.06	7.97 ± 1.12	3.91 ± 1.49	154.09 ± 36.30	69.67 ± 11.94	52.96 ± 29.27
	4-year	15.15 ± 1.87	9.66 ± 1.46	4.70 ± 1.34	208.31 ± 44.20	88.44 ± 16.73	74.41 ± 11.14
	11-year	16.29 ± 0.95	10.14 ± 1.99	4.80 ± 1.97	226.80 ± 43.75	97.61 ± 29.71	78.88 ± 26.84
*LSD*_0.05_		4.95	3.34	3.09	117.7	50.31	38.01

### Soil NH_4_^+^-N and NO_3_^−^-N concentrations

**Figure 2:**
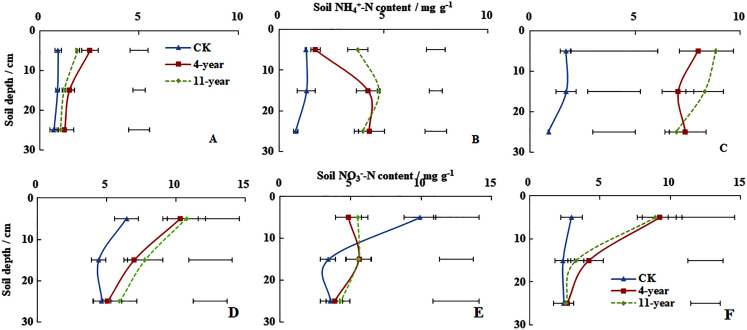
Soil NH_4_^+^-N and NO_3_^−^-N concentrations (mean ± standard deviation, *n* = 3). For the F (fallow control), 4-year old and 11-year old lucerne grasslands on 28 November 2013 (A, D), 7 January 2014 (B, E) and 6 March 2014 (C, F). Bars represent the least significant difference LSD (*P* = 0.05) for values of soil NH_4_^+^-N concentration at each depth.

On 28 November soil NH_4_^+^-N concentrations at a depth of 0–300 mm remained below five mg g^−1^ for all three treatments (Fig. 2A). Higher concentrations of soil NH_4_^+^-N were measured at a depth of 0–100 mm, which followed the ranking of 4-year-old grassland > 11-year-old grassland > F (*P* < 0.05). Soil NH_4_^+^-N concentrations at a depth of 0–300 mm for the three treatments were also <5 mg g^−1^ on 7 January (Fig. 2B). Higher soil NH_4_^+^-N concentrations were measured at a depth of 100–200 mm, which followed the rankings of 11-year-old grassland > 4-year-old grassland > F with values of 4.77, 4.27, and 1.31 mg g^−1^, respectively (*P* < 0.05). On 6 March, soil NH_4_^+^-N concentrations at a depth of 0–300 mm were only <5 mg g^−1^ for F (Fig. 2F). Concentrations measured at a depth of 0–100 mm were all higher at 1.70, 7.99, and 8.84 for F, 4-year-old and 11-year-old grasslands, respectively.

On 28 November, soil NO_3_^−^-N concentrations for the three treatments at a depth of 0–100 mm followed the order of 11-year-old grassland > 4-year-old grassland > F with values of 10.77, 10.34, and 6.46 mg g^−1^, respectively (Fig. 2D, *P* < 0.05). Soil NO_3_^−^-N contents at a depth of 0–300 mm for the three treatments remained <10 mg g^−1^ on January 7 and higher values were measured at a depth of 0–100 mm for the 11-year-old grassland and the F treatment (Fig. 2E). Soil NO_3_^−^-N concentrations at a depth of 0–100 mm followed the order of F > 4-year-old grassland > 11-year-old grassland with values of 9.92, 5.50, and 4.86 mg g^−1^, respectively (*P* < 0.05). On 6 March, soil NO_3_^−^-N concentrations at a depth of 0–300 mm remained <10 mg g^–1^ for all three treatments (Fig. 2F). In the 0–100 mm layer, values were 2.98, 9.24, and 8.93 for the F, 4-year-old, and 11-year-old grassland, respectively.

### Numbers of nitrification and denitrification microbes

The MPN method was used to estimate changes in the numbers of nitrifying and denitrifying organisms throughout the freeze/thaw cycles (Table 3). Highest MPN values of nitrosobacteria of approximately 9,380 were found in the F treatment on 28 November (*P* < 0.05). The highest MPN value for nitrifying bacteria of 15,970 was observed in the 4-year-old grassland on 28 November (*P* < 0.05). The maximum value of 31,470 denitrifiers per unit of soil dry mass was found in the 4-year-old grassland on 6 March (*P* < 0.05). The number of MPN denitrifiers in the 11-year-old grassland was 15,550 on 28 November (*P* < 0.05).

**Table 3:** Most probable numbers (MPN, ×10^3^ g^−1^) of nitrosobacteria (Nitrite), nitrifying bacteria (Nitrate) and denitrifying bacteria (Denitrifying) estimated for the soil at a depth of 0–100 mm for the three measurement periods.

Date	Treatment	Nitrite	Nitrate	Denitrifying
28 November 2013	F	9.38 ± 2.38	12.77 ± 3.80	14.18 ± 2.54
	4-year	6.35 ± 2.16	15.97 ± 2.17	19.39 ± 2.11
	11-year	3.62 ± 1.30	11.96 ± 2.88	15.55 ± 4.60
7 January 2014	F	3.53 ± 1.33	15.50 ± 2.11	23.30 ± 7.28
	4-year	8.41 ± 1.57	15.55 ± 1.83	23.68 ± 4.88
	11-year	3.43 ± 1.50	10.31 ± 2.63	28.00 ± 5.35
6 March 2014	F	6.80 ± 1.38	14.22 ± 5.70	18.88 ± 6.96
	4-year	7.67 ± 2.30	11.62 ± 3.97	31.47 ± 8.88
	11-year	4.84 ± 0.48	13.75 ± 3.72	20.30 ± 5.61
*LSD*_0.05_		3.57	3.57	12.05

**Note:**

For the F (fallow control), 4 and 11-year old lucerne crops. Data shown are Mean ± standard deviation, *n* = 3.

### Factors influencing N_2_O emissions

Data from the different treatments were analyzed to estimate the impact of freeze-thaw cycles on N_2_O emissions, and to remove additional factors such as the age of lucerne grasslands and nitrogen fixing capacity. The correlation matrix at a depth of 0–100 mm (Table 4) indicated that N_2_O emissions were significantly correlated with soil water content, soil temperature, NH_4_^+^-N concentrations, and the number of nitrosobacteria and denitrifying bacteria. MBN was positively correlated with MBC (*P* < 0.01). The NH_4_^+^-N concentration was also correlated with soil water content (*P* < 0.05). Further, the numbers of nitrosobacteria and denitrifying bacteria were related to soil temperature (*P* < 0.05). The number of denitrifying bacteria was significantly correlated with the soil NH_4_^+^-N concentration (*P* < 0.05). The correlations between N_2_O emissions and these variables at depths of 100–200 and 200–300 mm were similar to those for the 0–100 mm depth.

**Table 4:** Correlation matrix of N_2_O fluxes, soil properties and microbial numbers in the 0–100 mm soil depth.

	N_2_O	W	T	MBC	MBN	MBC/MBN	MBC/SOC	MBN/N	NH_4_^+^-N	NO_3_^−^-N	nitrite	nitrate
W	−0.34*	1										
T	−0.50**	0.91**	1									
MBC	−0.07	0.09	0.08	1								
MBN	−0.13	0.33*	0.20	0.50**	1							
MBC/MBN	0.05	−0.14	−0.05	0.60**	−0.34*	1						
MBC/SOC	−0.11	0.07	0.08	0.46*	0.12	0.37*	1					
MBN/N	−0.17	0.20	0.24	0.24	0.64**	−0.27	0.01	1				
NH_4_^+^-N	0.33*	0.38*	0.13	−0.14	0.26	−0.33*	−0.36*	−0.04	1			
NO_3_^−^-N	0.19	0.11	0.19	0.42*	0.03	0.41*	0.47**	−0.05	0.03	1		
nitrite	−0.35*	0.29	0.35*	−0.26	−0.12	−0.14	−0.08	0.18	0.07	−0.21	1	
nitrate	−0.26	−0.08	0.073	−0.17	−0.23	−0.03	−0.04	−0.01	−0.24	−0.07	−0.07	1
denitrifying	0.37*	−0.19	-0.34*	−0.19	0.06	−0.27	−0.01	−0.17	0.43**	0.05	−0.17	−0.16

**Note:**

Where W is soil water content, T soil temperature, MBC microbial biomass C, MBN microbial biomass N, SOC soil organic carbon, N total nitrogen, NO_3_^−^N concentration of soil nitrogen as nitrate, NH_4_^+^ concentration of soil nitrogen as ammonium, nitrite represents the number of nitrosobacteria, nitrate represents the number of nitrifying bacteria and denitrifying represents the number of denitrifying bacteria (*n* = 27). * *P* < 0.05; ** *P* < 0.01.

Autocorrelated relationships in the matrix were analyzed to identify the main factors that contribute to N_2_O emissions throughout freeze/thaw cycles. The results of PCA for soil variables at depths of 0–100 mm are shown in Fig. S2. PCA showed that N_2_O emissions were dominantly attributable to the number of denitrifying bacteria during the freeze/thaw period. The number of nitrosobacteria, soil temperature, and soil NH_4_^+^-N concentration were dominant variables to explain differences in N_2_O emissions.

## Discussion

### Diurnal dynamics of N_2_O emissions during the freeze/thaw cycle

The highest daily average N_2_O emission was 0.3522 mg m^−2^ h^−1^ for the 11-year-old grassland on 28 November (Fig. 1A). Increased emissions with increasing cultivation age are likely attributable to different N and C concentrations in both plant and soil (Wang et al., 2014b). The better developed root system in older grasslands produces more residue and litter and secrets more SOC, TN, and organic acids (Hong, 2009), thus increasing the potential for N_2_O production (Rochette & Janzen, 2005). Further, the mean N_2_O emissions for the 11-year-old grassland on 7 January were higher than average emissions during the growing season (Rochette & Janzen, 2005). The maximum N_2_O emission for the 11-year-old grassland (0.0879 mg m^−2^ h^−1^) in the freeze/thaw cycle was significantly higher than the maximum value (0.0323 mg m^−2^ h^−1^) during the rest of the growing season.

Increased soil temperature during the daytime enhanced soil water content, thus limiting soil gas diffusion (Müller et al., 2002) and leading to reduced N_2_O emissions (Burton & Beauchamp, 1994). The low variability in N_2_O emissions between 11:00 and 14:00 (Fig. 1) may be explained by the diurnal dynamics of soil temperature and soil water content. High values of soil water content for the 11-year-old grassland on 6 March were associated with low emissions (Fig. 1). High emissions between 18:00 and 22:00 could be explained by changes in the soil volume following freezing and the release of trapped N_2_O into the atmosphere, which had accumulated in the frozen soil (Burton & Beauchamp, 1994).

Negative N_2_O emissions were measured on a number of ocassions (Fig. 1), which is consistent with other findings (Chapuis-lardy et al., 2007). Negative fluxes usually indicate that N_2_O is reduced and emitted as N_2_, representing the final step of the microbial denitrification process. Anaerobic conditions could stimulate N_2_O consumption by soils resulting in the conversion of N_2_O to N_2_, with little mineral N and readily available organic matter as energy sources for denitrifying activity of microbes (Bremner, 1997; Chapuis-lardy et al., 2007).

### Influence of factors on N_2_O emissions during freeze/thaw cycles

Changes in soil temperature and soil water content affect N_2_O emissions by influencing metabolic activities of microorganisms, soil aeration, substrate availability, and nutrient redistribution (Wagner-Riddle et al., 2017). The N_2_O/N_2_ emissions ratio has been shown to decrease with increasing temperature (Holtan-Hartwig, Dörsch & Bakken, 2002). At the tested site, the correlation between soil temperature and N_2_O emissions was negative (Table 4, Fig. S2). Other findings also identified soil temperature, rather than soil water content, as more important for regulating N_2_O emissions (Koponen & Martikainen, 2004; Luo et al., 2013). This is likely attributable to the effects of soil temperature on enzymatic activity and substrate limitation (Stark & Firestone, 1995). Soil temperature has also been shown to affect the osmotic status of microbial cells, the rates of gas diffusion, and soil water content (Wu et al., 2013). Although the findings of the present study showed that soil water content was a significant factor influencing N_2_O emissions in freeze/thaw cycles, soil temperature could better explain N_2_O emissions and microbial numbers (Table 4, Fig. S2).

Soil freeze-thaw cycles exert profound impacts on soil physical structure, solute distribution, and nutrient availability. N_2_O reductase activity is important for regulating N_2_O emissions during freeze/thaw cycles, which is strongly influenced by the availability of decomposable organic matter (Wang et al., 2014a) as well as C and N sources (Christensen & Christensen, 1991; Müller et al., 2002). The present study showed that MBC, MBN, and the MBC/SOC ratio were positively correlated with N_2_O emissions (Table 4). Although other research has found increases in the availability of carbon from fragmented organic matter or dead microorganisms during freeze/thaw cycles (Christensen & Christensen, 1991; Wang et al., 2014a), the results obtained in the present study showed that MBC concentration did not significantly change during the freeze/thaw cycles. A probable explanation is that MCB was consumed by microbes that survived the freeze/thaw cycles. A significantly positive correlation was found between MBN and MNC (Table 4), which agrees with previous findings (Gou et al., 2015).

The soil NH_4_^+^-N concentration increased during freeze/thaw cycles, whereas the soil NO_3_^−^-N concentration decreased significantly (Fig. 2). This increase in NH_4_^+^-N concentration is likely attributable to the disturbance of the topsoil structure because of freeze/thaw cycles and the accelerated soil nitrogen mineralization, influenced by soil water content and temperature (Song et al., 2017). Soil water concentration was positively correlated with soil NH_4_^+^-N concentration (Table 4). The reduction in soil NO_3_^−^-N content during freeze/thaw cycles can be explained by low N uptake, leading to high rates of denitrification and N_2_O production (Pu, Saffigna & Strong, 1999). Soil NO_3_-^−^N as the direct source of N_2_O decreased during the period when N_2_O emissions are highly variable (Table 4, Figs. 1 and 2).

The number of nitrosobacteria was linked to soil temperature but not to soil water content (Table 4). Potential explanations are that nitrification depends on soil water content (Stark & Firestone, 1995) or that soil temperature is the limiting factor during freeze/thaw cycles. Ammonia-oxidizing bacteria are essential for nitrification and freeze/thaw cycles as these influence the number of the nitrifying organisms (Tzanakakis, Taylor & Bottomley, 2020). However, the correlations between N_2_O emissions and the numbers of nitrifying bacteria were not significant (Table 4). Similarly, although a positive correlation was found between the numbers of nitrosobacteria and nitrification substrates (NH_4_^+^-N), as well as a negative correlation between the numbers of nitrosobacteria and nitrification products (NO_3_^−^-N), these correlations were not significant. A possible explanation is that the activities of nitrifying microorganisms or nitrification might be diminished during freeze/thaw cycles (Papen & von Berg, 1998).

Nitrification is known to be the largest source of N_2_O emissions in semi-arid regions where soils are rarely sufficiently anaerobic to induce denitrification (Wagner-Riddle et al., 2017). However, freeze/thaw cycles induce N_2_O emission because of the limitation of the supply of oxygen needed for nitrification (Goreau, Kaplan & Wofsy, 1980). Thawing of the top soil layer and the creation of anaerobic conditions resulting from water saturation would induce denitrification (Müller et al., 2002; Dusenbury et al., 2008; Wu et al., 2013). In the present study, N_2_O emissions were significantly linked to the number of denitrifying bacteria (Table 4). Also, the number of denitrifying bacteria in the tested soils was significantly greater than the number of nitrifying microorganisms (Table 3). This is consistent with the findings of Holtan-Hartwig, Dörsch & Bakken (2002) who suggested that freeze-thaw cycles enhance denitrification. Peterjohn (1991) found that denitrifying enzymes can survive under drought and can be reactivated when soil water content increased. Also, soil water saturation stimulated soil microbial activity, restricted the oxygen supply, and increased CO_2_ concentrations in soil (Chamindu et al., 2019), all of which could stimulate denitrification.

Large diurnal amplitudes in surface soil temperature are characteristic for the Loess Plateau, resulting from thawing during the daytime and refreeze at night (Figs. 1D–1F). This favors the coupling of nitrification and denitrification (Müller et al., 2002), which could have both contributed to N_2_O production in the present study. Here, both the number of denitrifying bacteria and nitrosobacteria bacteria were found to correlate significantly with N_2_O emissions, which is in general agreement with prior findings.

Interpretations of this study are restricted by data availability. In particular, the authors are aware of the importance of the soil microclimate (*i.e*., temperature and water content) and microbial activities throughout the year; however, the present study is limited because of a lack of such data. Thus, broader interpretations should be made with caution. Moreover, further studies of freeze/thaw cycles in the field with higher frequency of N_2_O measurement are warranted.

## Conclusions

This study showed that during freeze/thaw cycles, N_2_O emissions from a 11-year-old grassland were higher than those from a 4-year-old grassland. Freeze/thaw cycles increase total N_2_O emissions from lucerne grasslands and strongly affect the variability in N_2_O emissions. These findings confirm that the dynamics of the number of denitrifying bacteria are most important for regulating N_2_O emissions from lucerne grasslands during freeze/thaw cycles on the Loess Plateau. The effects of changes in the number of nitrosobacteria, soil temperature, and NH_4_^+^-N content were weaker. This suggests the potential for reducing N_2_O emissions during freeze/thaw cycles if the amplitude of the dynamics of soil temperature, which influences the number of denitrifying bacteria and nitrosobacteria, can be stabilized. Confirmation of this suggestion requires further research.

## Supplemental Information

10.7717/peerj.12216/supp-1 Supplemental Information 1 Principal component analysis of soil factors influencing N_2_O emission flux at soil depths 0-100 mm.

10.7717/peerj.12216/supp-2 Supplemental Information 2 Raw Data.

## Additional Information and Declarations

### Data Availability

The following information was supplied regarding data availability: The raw data is available as a Supplementary File.
