# Supplementary material for: Freezing and thawing cycles affect nitrous oxide emissions in rain-fed lucerne (Medicago sativa) grasslands of different ages
Source: PeerJ. 2021 Oct 5;9:e12216. doi: 10.7717/peerj.12216 (PMC8501990; doi:10.7717/peerj.12216)
Supplement: Supplemental Information 1 [file peerj-09-12216-s001.docx]

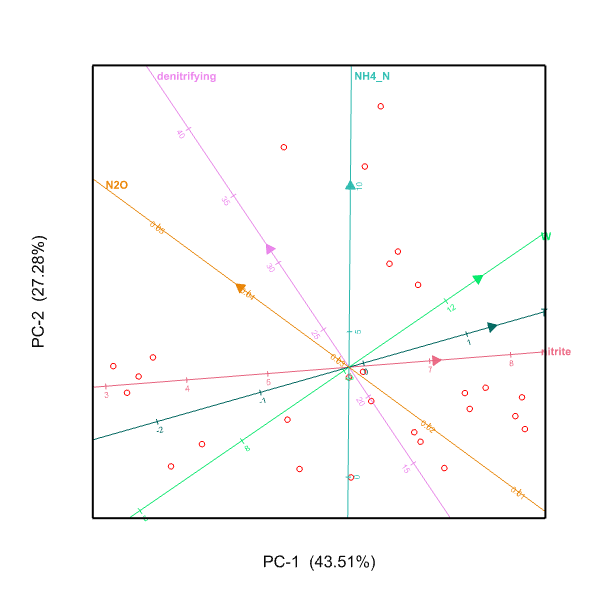


**Figure S1** Principal component analysis of soil factors influencing N_2_O emission flux at soil depths 0-100 mm. The loadings of soil factors are shown as vectors. W is soil water content, T soil temperature, NH4-N soil nitrogen as ammonium concentration NH_4_^+^-N concentration, nitrite represents the number of nitrosobacteria and denitrifying represents the number of denitrifying bacteria. (n=27)
